# Improving the Micropore Capacity of Activated Carbon by Preparation under a High Magnetic Field of 10 T

**DOI:** 10.1038/s41598-019-43818-y

**Published:** 2019-05-16

**Authors:** Atom Hamasaki, Ayumi Furuse, Yuya Sekinuma, Kazuki Fujio, Masashi Iide, Sumio Ozeki

**Affiliations:** Faculty of Science, Shinshu University, Matsumoto, Nagano 390-8621 Japan

**Keywords:** Porous materials, Structural properties

## Abstract

The influence of an applied magnetic field on the formation of carbon materials from coal tar pitch is investigated. Under an applied magnetic field, crystallites in a mesophase resembling liquid crystals are magnetically oriented during the carbonization process. Compared with that under a nonmagnetic field, carbonized coal tar pitch under a strong magnetic field of 10 T, generated by a superconducting magnet, has a highly oriented structure of carbon crystallites. The orientation of samples prepared under 2 T, which can easily be supplied by an electromagnet, was insufficient. Activation by potassium hydroxide is effective for affording a precursor for activated carbon. The activated carbon obtained under a strong magnetic field has a unique adsorption ability, which arises from its increase in relative surface area and total pore volume compared with those of an activated carbon sample prepared from a precursor produced under zero magnetic field. The precursor carbonized under a magnetic field of 10 T contains a larger number of crystallites than that carbonized under a 0-T magnetic field, which leads to high-performance activated carbon.

## Introduction

Carbon materials, such as activated carbon and graphite, are very useful functional materials. For example, carbon materials are important in applications such as gas separation using pressure swing adsorption, water purification, air cleaning, electrodes in metal refining, pencils, and lubricants. Recently, nanocarbon, for example, carbon nanotubes and graphene, has attracted attention, but conventional carbon materials have still been applied and used in advanced technology, such as activated carbon in electric double-layer capacitors and carbon fibre-reinforced plastic for the wings of airplanes. Carbon materials are becoming increasingly important in our lives; therefore, the amount of carbon materials that is produced will need to increase to meet the demand. The carbon source, preparation conditions such as temperature and atmosphere, and preparation methods have an important influence on the properties of carbon materials because the structure of carbon materials controls their properties^1,2^. The discovery of new controlling methods will lead to the refinement of carbon materials as advanced functional materials.

**Figure 1:**
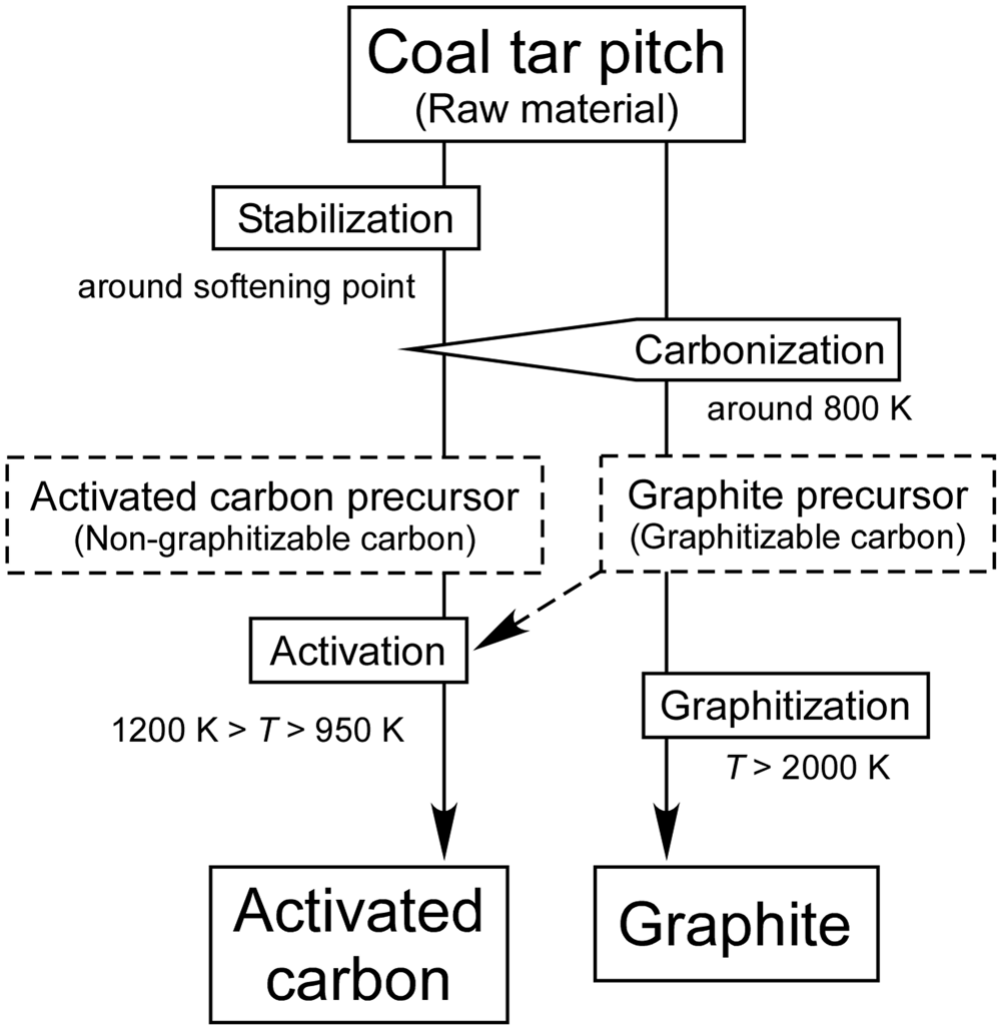
General preparation method of activated carbon and graphite from coal tar pitch.

Coal tar pitch, which is a mineral and contains not only carbon but also other elements such as nitrogen, oxygen, and sulfur, has been used as a raw material for carbon products, including both activated carbon and graphite. A general preparation method for activated carbon and graphite from coal tar pitch is shown in Fig. 1^3^. Graphite is prepared by thermal treatment processes, which can be separated into the crystallite growth process in an unreactive atmosphere at less than 1200 K (carbonization) and subsequent crystallite connection to make large graphene sheets at up to 4000 K (graphitization). A carbon hexagonal layer grows well during the carbonization process and is called the graphite precursor (graphitizable carbon). If a raw material is stabilized in an oxidative atmosphere around its softening point, because oxygen acts as a cross-linker between carbon crystallites, the growth of hexagonal carbon is inhibited during thermal treatment above the stabilization temperature. As a result, the base of each pore is constructed of crystallites. This carbon material is called the activated carbon precursor (non-graphitizable carbon).

Magnetic fields will interact with any material regardless of its magnetism, but because of the angular momentum of electrons, magnetic fields act weakly on diamagnetic materials. In the 1990s, helium (He)-free superconducting magnets were developed, and this advancement has led to dramatic progress in research. Many of the examples of orientation in the direction of a magnetic field of large molecules, such as polymers^4–6^ and large domains consisting of small molecules, such as liquid crystals^7–9^ and lipid membranes^10–13^, were discovered by using He-free superconducting magnets.

Because the anisotropic structure of hexagonal layers is composed of sp^2^ bonds, carbon materials may also lead to the magnetic orientation of graphene domains. When a pitch-based carbon material is heat-treated in an unreactive atmosphere, it passes through the liquid phase in the process of carbonization. The viscosity of the raw material is extremely high, and the material after carbonization is a solid; however, its viscosity greatly decreases at approximately 600–800 K^14^. At these temperatures, spherulites are generated, and they are in the mesophase, which has properties similar to liquid crystals^15^. In 2012, we constructed a new furnace system with a superconducting magnet and applied a magnetic field to the heat-treatment process of carbon materials. Compared with that of a sample produced under a 0-T magnetic field, the intensity of the (002) diffraction peak in X-ray diffraction (XRD) profiles^16^ of heat-treated coal tar pitch samples with no stabilization, which is the same as a graphite precursor prepared under a magnetic field of 10 T, increased by approximately 30% despite the crystallites having the same dimensions^17^. This magnetic field effect was explained by the magnetic orientation arising from the anisotropic structure of hexagonal layers composed of sp^2^ bonds. Although anomalous birefringence of mesophase pitch prepared under magnetic fields smaller than 1 T was reported in the 1980s^18–21^, it became clear from our study that the orientation degree was greatly improved upon increasing the magnetic field from 3 T to 6 T^17^. The magnetic orientation energy *E*_r_ of a domain composed of *N* molecules under magnetic flux density *B* is given by^22,23^:

1 $${E}_{{\rm{r}}}=-\frac{N{\rm{\Delta }}\chi \,{B}^{2}}{2{\mu }_{0}}{\cos }^{2}\theta ,$$

2 $${\rm{\Delta }}\chi ={\chi }_{\perp }-{\chi }_{//}=3({\chi }_{{\rm{M}}}-{\chi }_{//}),$$

3 $${\chi }_{{\rm{M}}}=({\chi }_{\perp }+2{\chi }_{//})/3.$$

Here, *μ*_0_ is the magnetic permeability in vacuum, and *θ* is the angle between the direction of a magnetic field and the easy axis of magnetization. The magnetic anisotropy Δ*χ*^22^ is the difference between perpendicular (*χ*_⊥_) and parallel (*χ*_//_) magnetic susceptibilities, as illustrated in Eq. (2), while the molar magnetic susceptibility *χ*_M_ is determined using Eq. (3). The plane of the benzene ring is assigned as *χ*_//_, which aligns with the easy axis of magnetization, and the direction perpendicular to the ring is *χ*_⊥_, which aligns with the hard axis of magnetization. Since a *B*^2^ effect is expected, it is reasonable that the magnetic flux density increases sharply at 3 T or above. However, the magnetic field effect was saturated above 6 T. The crystallite size estimated from the XRD results was 2.9 × 2.9 × 2.7 nm^3^. If we assumed that only a layered graphene structure was obtained, the anisotropic magnetic susceptibility was calculated as Δ*χ* = −8.6 × 10^−7^ m^3^ mol^−1^^17^. Since the thermal energy was approximately 6.6 kJ mol^−1^ at 793 K, it was calculated that the magnetic orientation could be sufficiently complete at more than 6 T by overcoming Brownian motion, with only approximately 4000 crystallites moving cooperatively. In fact, the crystallite contained other atoms, such as oxygen and sulfur; therefore, the actual value of anisotropic susceptibility was slightly smaller than the ideal value, and it seemed that more crystallites moved cooperatively. Nevertheless, considering the size of the mesophase microspheres (diameter on the micrometre scale), this result was quite reasonable. We called this material a highly oriented carbonized pitch (HOCP) as opposed to general carbonized pitch (GCP).

There have been many precedents for preparing composite materials while orienting carbon materials, such as graphene and nanotubes, in a magnetic field. However, applying a strong magnetic field to the preparation process of a functional carbon material to drastically change the structure has never been reported. In the days when only electromagnets could be used, the strength of the magnetic field was lower than that provided by modern equipment. Additionally, to prepare the material, the distance between the poles of an electromagnet would be insufficient. Preparation by using a superconducting magnet may be expected to be beneficial not only for the application of a high magnetic field but also for the large preparation space compared with that of an electromagnet. We attempted to prepare a high-performance carbon material in this study by preparing activated carbon from HOCP.

## Results and Discussion

### Optimization of magnetic field effects on carbonized process

**Figure 2:**
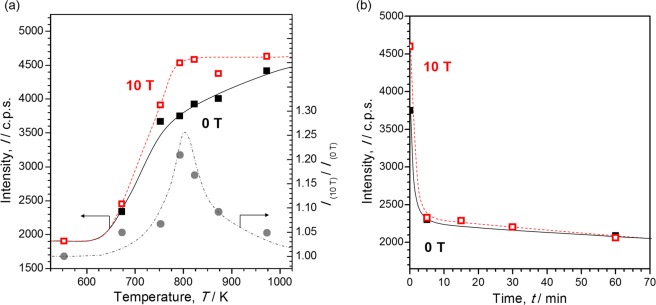
(**a**) Heat-treatment-temperature dependence of the (002) XRD peak intensity of carbon materials prepared in the absence (solid line, closed squares) and presence (dashed line, open squares) of a 10-T magnetic field. Relative value of the intensities of the (002) XRD peaks at 10 and 0 T, *I*_(10T)_/*I*_(0T)_ (dashed-dotted line, circles). (**b**) Stabilization time dependence of the (002) XRD peak intensity of activated carbon precursors prepared in the absence (solid line, closed squares) and presence (dashed line, open squares) of a 10-T magnetic field.

To clarify the optimal temperature for the magnetic field effect, we carried out only the carbonization treatment of coal tar pitch with the temperature varied from 553 K to 973 K (see Fig. S1(a), process A), which was based on the general method for preparing a graphite precursor. The intensity of the XRD peak at 26°, assigned to the (002) plane of carbon hexagonal layers prepared in the absence and presence of a 10-T magnetic field, at each temperature is plotted in Fig. 2(a). Carbonization did not progress below 650 K, so naturally, no magnetic field effect was observed in this temperature region. Carbonization occurred gradually from 650 K until approximately 1000 K under zero magnetic field. In contrast, the (002) peak intensity of the samples prepared under a 10-T magnetic field increased rapidly with increasing heat-treatment temperature until 800 K and then became saturated above this temperature. The difference between the (002) XRD peak intensities of carbon materials prepared in the absence and presence of the 10-T magnetic field, denoted by *I*_(10T)_/*I*_(0T)_, is also shown in Fig. 2(a). The maximum magnetic field effect was observed at approximately 800 K. We considered this finding as evidence for a magnetic field effect caused by magnetic orientation because this is the temperature at which the mesophase of coal tar pitch, which is a liquid crystal-like structure containing carbon crystallites, appears.

We have reported previously that it was impossible to obtain the magnetic field effect for a general activated carbon precursor prepared with stabilization^17^. This result was explained by the oxygen cross-linked structures that formed in the carbon material during thermal treatment in an oxidative atmosphere^2^; i.e., the formation of an oxygen cross-linked structure by stabilization not only led to suppression of carbonization but also made liquid crystal-like behaviour impossible. This was considered the reason why the magnetic field effect did not appear. To elucidate the time required for cross-linked structure formation, stabilization was carried out before carbonization with the time varied from 0 to 120 minutes (see Fig. S1(a), process B). Here, preparation methods other than 0 minutes were based on the general method for preparing an activated carbon precursor. The (002) XRD peak intensity was plotted against stabilization time as illustrated in Fig. 2(b). The (002) diffraction drastically decreased after stabilization for just 5 minutes before carbonization regardless of the absence or presence of a magnetic field. Under oxidizing conditions, cross-linking proceeds regardless of the presence or absence of a magnetic field. A cross-linked structure was effectively constructed by slight stabilization, and no magnetic field effect was observed in only 5 minutes. We concluded that no stabilization was the only way to obtain highly oriented anisotropic carbon from coal tar pitch. We would like to discuss the difference in the structure and function of GCP and HOCP prepared in the absence and presence of 10 T at 793 K without stabilization (as process A), which are described as P-GCP and P-HOCP below.

### Specific structure of carbonized pitch under a high magnetic field of 10 T

**Figure 3:**
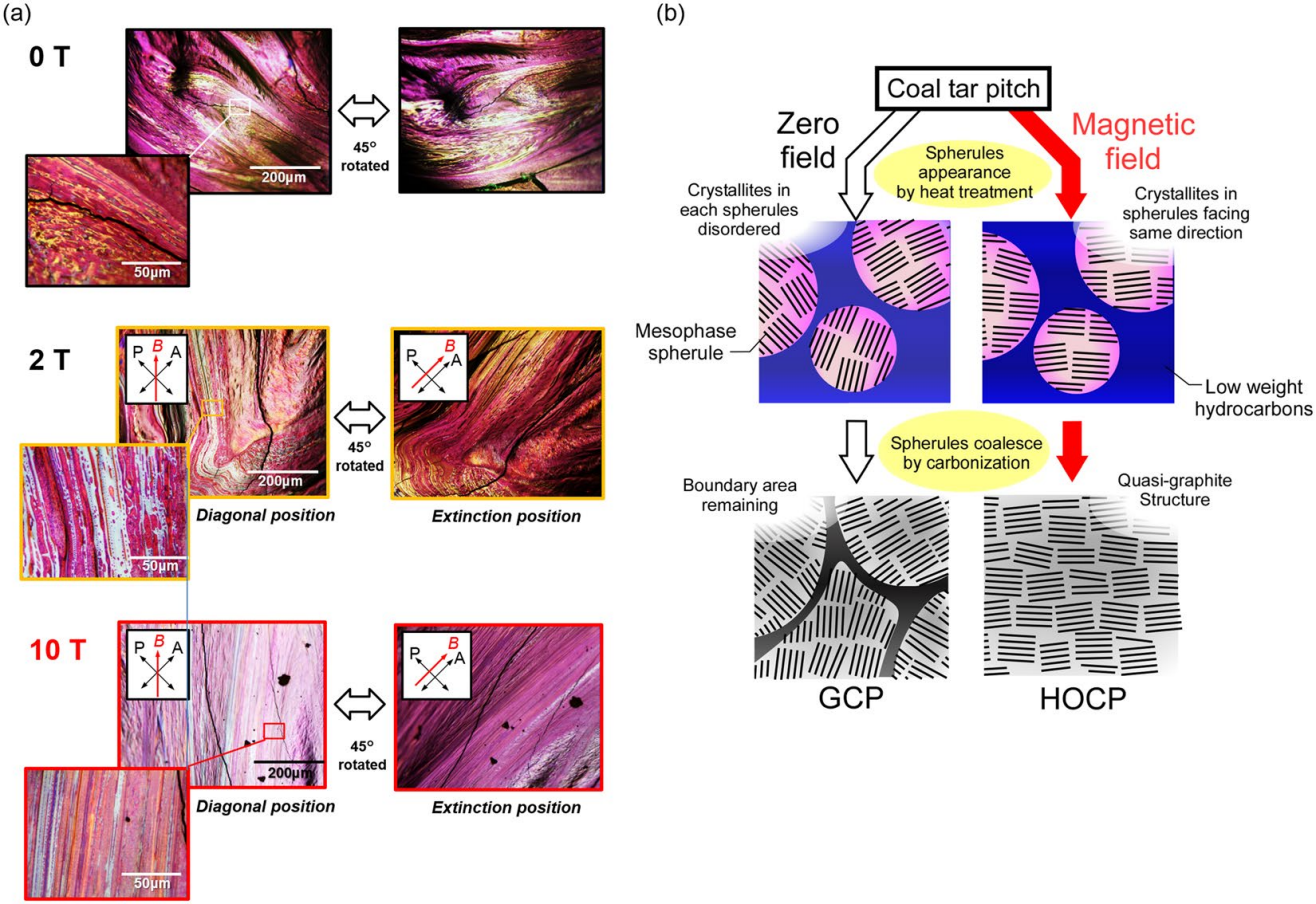
(**a**) Polarizing microscopy image of carbonized coal tar pitch prepared in the absence and presence of magnetic fields of 2 T and 10 T. All figures show the diagonal position and extinction position. (**b**) Schematic drawing of a magnetic field effect on the carbonization process of coal tar pitch.

The oriented structures of P-GCP and P-HOCP were observed with a polarizing microscope. P-GCP had a flow-like structure derived from the fusion of the mesophase, as shown in Fig. 3(a)-0 T, which was confirmed by the polarizing microscope, and the two polarizers were set at the crossed Nicols position. As can be understood from the image rotated by 45° from the original position, the orientation direction varied over a wide region. In the magnified figure, isolated small mesophase spherules were observed, indicating that the fusion was not uniform. This carbon material corresponded to “general” anisotropic carbon because of the above; however, carbon crystallites were oriented only in a small number of mesophase units in the absence of a magnetic field. The direction of the crystallite was different between domains, as shown in Fig. 3(b). As a result, there were areas where the crystallite became sparse at the boundary. However, in the case of carbonization under a high magnetic field obtained by using a superconducting magnet, such as carbonization for P-HOCP, the magnetic orientation was markedly accelerated. As shown in Fig. 3(a)-10 T, the white region showing optical anisotropy was widely spread (diagonal position), and when the sample was rotated by 45°, the whole region became dark (extinction position). In contrast to the structure evident at 0 T, a fine domain structure was not observed, and the orientation degree was extremely high and had a highly uniform structure, both macroscopically and microscopically. We confirmed that P-HOCP exhibited a structure similar to the quasi-graphite structure, such as crystallites aligned over a wide range, as shown in Fig. 3(b). Although the orientation at approximately 2 T (which can be generated with a relatively strong electromagnet) was locally advanced, compared with that of 10 T, it was not complete, as shown in Fig. 3(a)-2 T. The structure of P-HOCP was a new structure obtained by using a strong magnetic field. If the magnetic orientation is complete at approximately 2 T, superconducting magnets will not be needed. However, the structures prepared in the presence of superconducting magnets were different in terms of the electromagnet level orientation. Although the required magnetic field strength depends on the raw material properties, the use of superconducting magnets is very important to provide new structures for carbon materials.

### Adsorption isotherms and pore distribution

**Figure 4:**
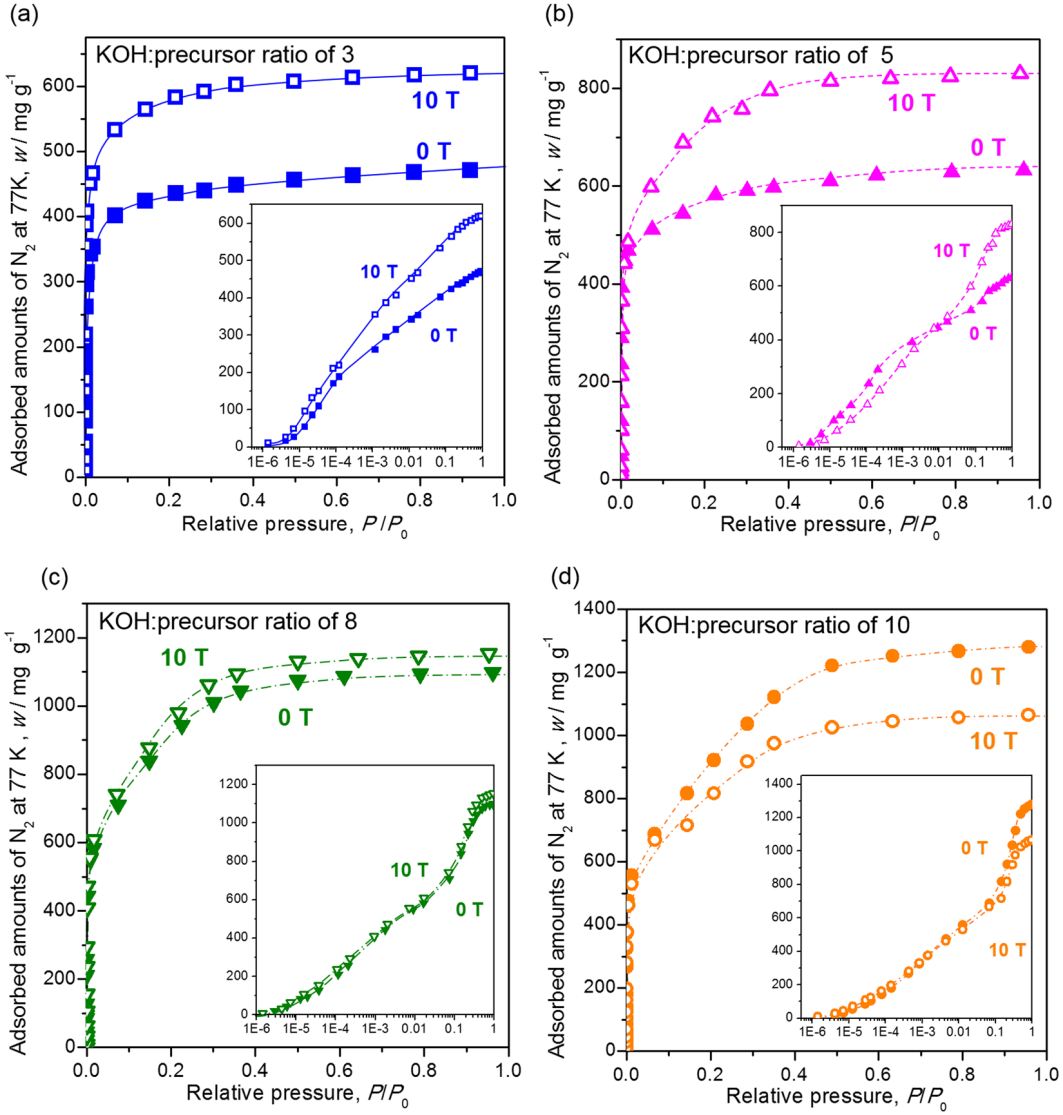
Adsorption isotherm of activated carbon prepared from P-GCP (closed marks) and P-HOCP (open marks) with a KOH:precursor ratio of (**a**) 3 (squares, solid line), (**b**) 5 (upward-pointing triangles, dashed line), (**c**) 8 (downward-pointing triangles, dashed-dotted line), (**d**) 10 (circles, dashed-double dotted line). Inset figures are the logarithmic plots.

Figure 4(a–d) display the adsorption isotherms of activated carbon prepared from P-GCP and P-HOCP with KOH:precursor ratios of 3, 5, 8 and 10, respectively. For activated carbon prepared from P-GCP and P-HOCP, micropores of less than approximately 2 nm diameter, which a Type I adsorbed isotherm according to IUPAC classification^24,25^ were formed in all samples except for that produced using a KOH:precursor ratio of 1. Table 1 shows the adsorption amount of each sample and the pore parameters calculated from the adsorption isotherms. Compared with that of activated carbon prepared from P-GCP, the adsorption amount of activated carbon prepared from P-HOCP increased by approximately 35% for a KOH:precursor ratio of 3. The adsorption amount of the sample prepared with a KOH:precursor ratio of 5 also increased by approximately 35%; however, its behaviour was different from that of the sample prepared with a KOH:precursor ratio of 3 at a relative pressure of less than 1 × 10^−4^. At a KOH:precursor ratio of 3, the adsorption amount of activated carbon prepared from P-HOCP was larger than that of activated carbon prepared from P-GCP at a relative pressure of less than 1 × 10^−4^. However, at a KOH:precursor ratio of 5, the adsorption amount in this region was smaller for activated carbon prepared from P-HOCP than activated carbon prepared from P-GCP. Although the number of relatively small to relatively large micropores increased evenly in P-GCP with an increasing amount of KOH, it was assumed that relatively large micropores were formed by enlarging a fine micropore in P-HOCP. In the case of a much larger KOH:precursor ratio, the superiority of applying a magnetic field was lost at a KOH:precursor ratio of approximately 8 and the magnetic field effect was completely inhibited at a KOH:precursor ratio of 10.

**Table 1:** Pore parameters of prepared activated carbon samples.

KOH ratio	Magnetic field (*B*/T)	Burn-off (%)	Adsorbed amount (*w*/mg g^−1^)	BET surface area (*A*_S_/m^2^ g^−1^)	Total pore volume (*V*_total_/cm^3^ g^−1^)	Micropore volume (*V*_micro_/cm^3^ g^−1^)	Micropore ratio (*V*_micro_/*V*_total_, %)	Pore size (*d*/nm)
1	0 T	—	20	61	0.026	0.025	96	—
	10 T	—	25	76	0.032	0.031	97	—
3	0 T	55	436	1275	0.55	0.51	93	0.78
	10 T	52	591	1697	0.74	0.71	96	0.78
5	0 T	62	592	1619	0.74	0.67	91	0.94
	10 T	52	798	2063	1.00	0.76	76	0.97
8	0 T	71	1065	2503	1.33	0.88	66	1.06
	10 T	65	1105	2623	1.38	0.96	69	1.05
10	0 T	71	1196	2443	1.49	0.98	66	1.19
	10 T	66	1003	2156	1.25	0.89	71	1.13

**Figure 5:**
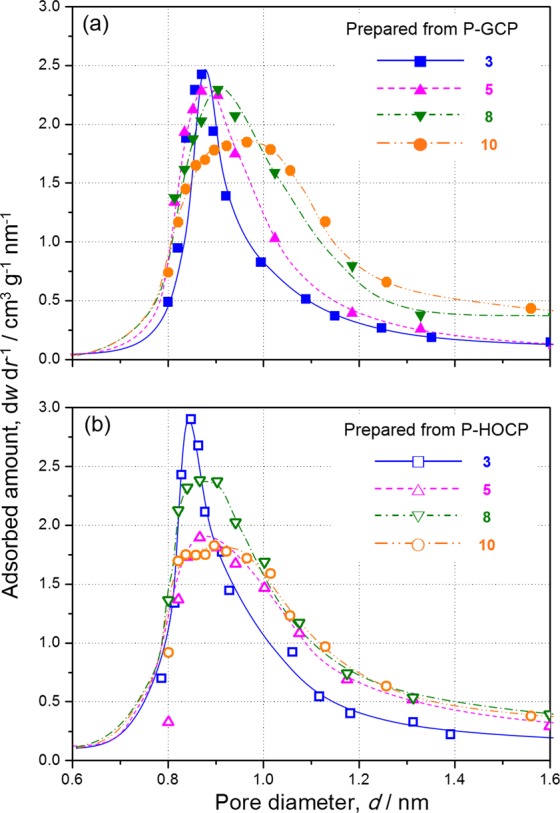
Pore size distributions of activated carbon prepared from (**a**) P-GCP (closed marks) and (**b**) P-HOCP (open marks) with KOH:precursor ratios of 3 (squares, solid line), 5 (upward-pointing triangles, dashed line), 8 (downward-pointing triangles, dashed-dotted line), and 10 (circles, dashed-double dotted line).

The pore distributions of activated carbon prepared from P-GCP and P-HOCP, as analysed by the Horvath–Kawazoe method^26,27^ from the obtained adsorption isotherms, are presented in Fig. 5(a,b), respectively. The pore sizes of activated carbon prepared from P-GCP and P-HOCP with a KOH:precursor ratio of 3 were 0.88 nm and 0.84 nm, respectively. The difference was extremely small, and most of the pores were distributed between 0.80 and 0.95 nm in both cases. However, it was observed that the adsorption amount of the activated carbon prepared from P-HOCP greatly increased in this region. The total adsorption amount in the range from 0.8 to 0.95 nm could be estimated to be approximately 1.25 times from the adsorption isotherm. When the KOH ratio was 5, the pore size distribution was shifted for both P-GCP and P-HOCP to expanding. In the case of P-GCP, pore sizes from 0.80 to 1.05 nm were mainly observed, but in the case of P-HOCP, the pore size was approximately 1.10 nm, which was slightly larger. From the viewpoint of the adsorption amount, the number of fine micropores with pore sizes between 0.8 and 0.9 nm was greatly reduced in P-HOCP compared to in P-GCP. This effect was reflected in the difference in isotherms between the KOH:precursor ratios of 3 and 5 at a relative pressure of less than 1 × 10^−4^. At KOH ratios of 8 and 10, the behaviour was completely different between P-GCP and P-HOCP. In the case of P-GCP, the pore size increased with increasing KOH:precursor ratio, whereas in the case of P-HOCP, the pore diameter hardly increased from that for the KOH:precursor ratio of 5.

### Mechanism of pore construction

**Figure 6:**
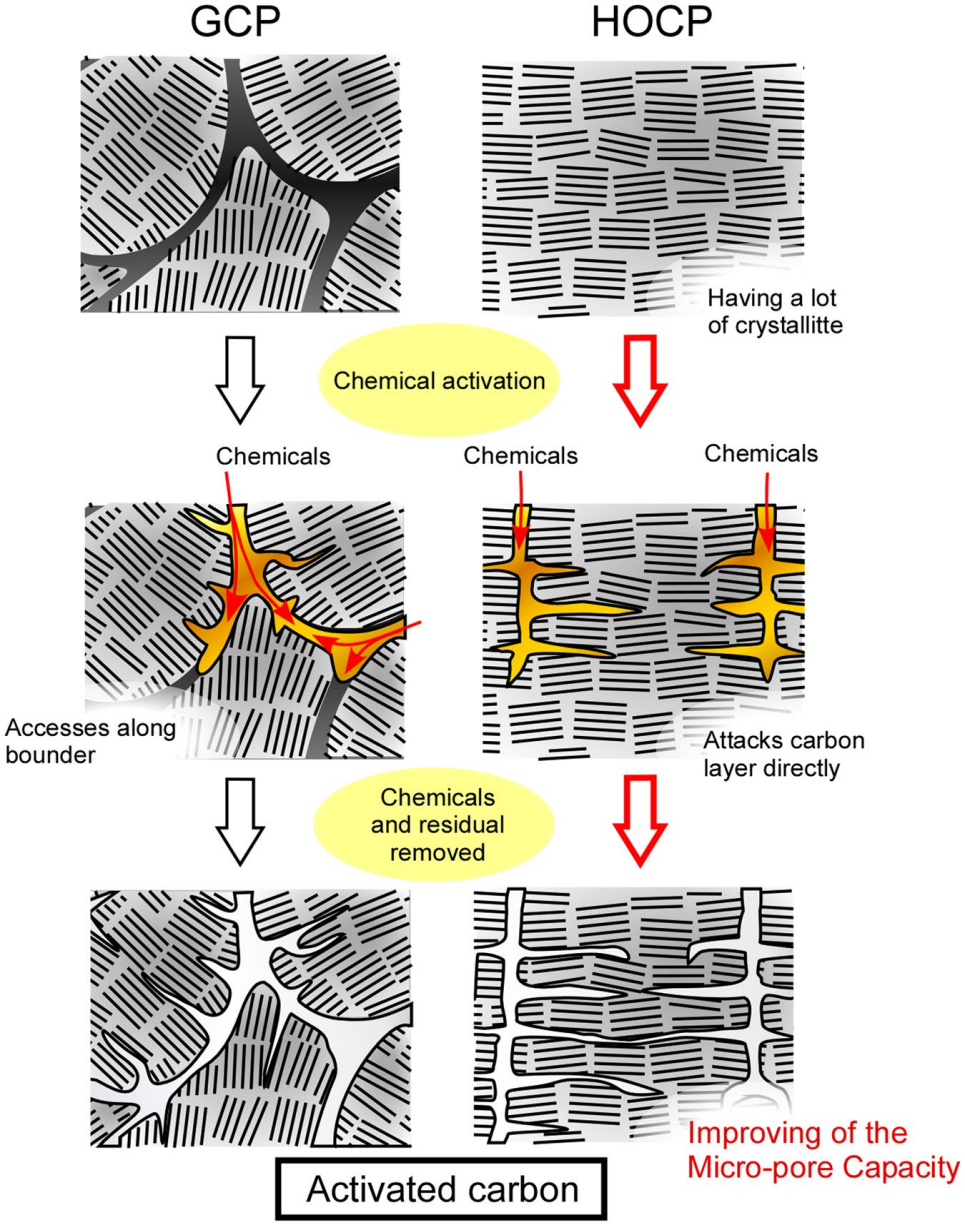
Schematic drawing of the effect of an applied magnetic field on the formation of activated carbon.

We predicted that these differences arose from the difference in the pore formation mechanism derived from the precursor structure of P-GCP and P-HOPC. To activate the carbon atoms that do not form part of the hexagonal layer of carbon to react, the activator atom must be located inside the structure. Therefore, an access pathway to these atoms is necessary for activation. Because P-GCP contained amorphous carbon derived from the fusion of mesophase spherules in random directions, as shown in Fig. 6, molecules were able to gain access along the amorphous carbon regions. Under an applied magnetic field, because oriented spherules coalesce, carbon hexagons form along the magnetic-field direction. This effect leads to a structure with no boundaries, that is, a graphene-like structure, which was also suggested from the results of the birefringence measurements, as shown in Fig. 2. As a result, P-HOCP does not contain access pathways because of the alignment of crystallites with the magnetic field; therefore, chemicals cannot easily gain access to the inter-crystallite and inter-carbon hexagonal layers^28^. The mechanism of chemical activation of HOPG proposed by Endo *et al*. involves metallic potassium penetrating into the crystallites with destruction of the hexagonal network to form the edge surfaces in graphite^29^ and chemicals intercalating into the interlayer of graphene plates. We propose that P-HOCP has a difficult structure for activation and that P-HOCP is activated in an activation mechanism similar to HOPG.

**Figure 7:**
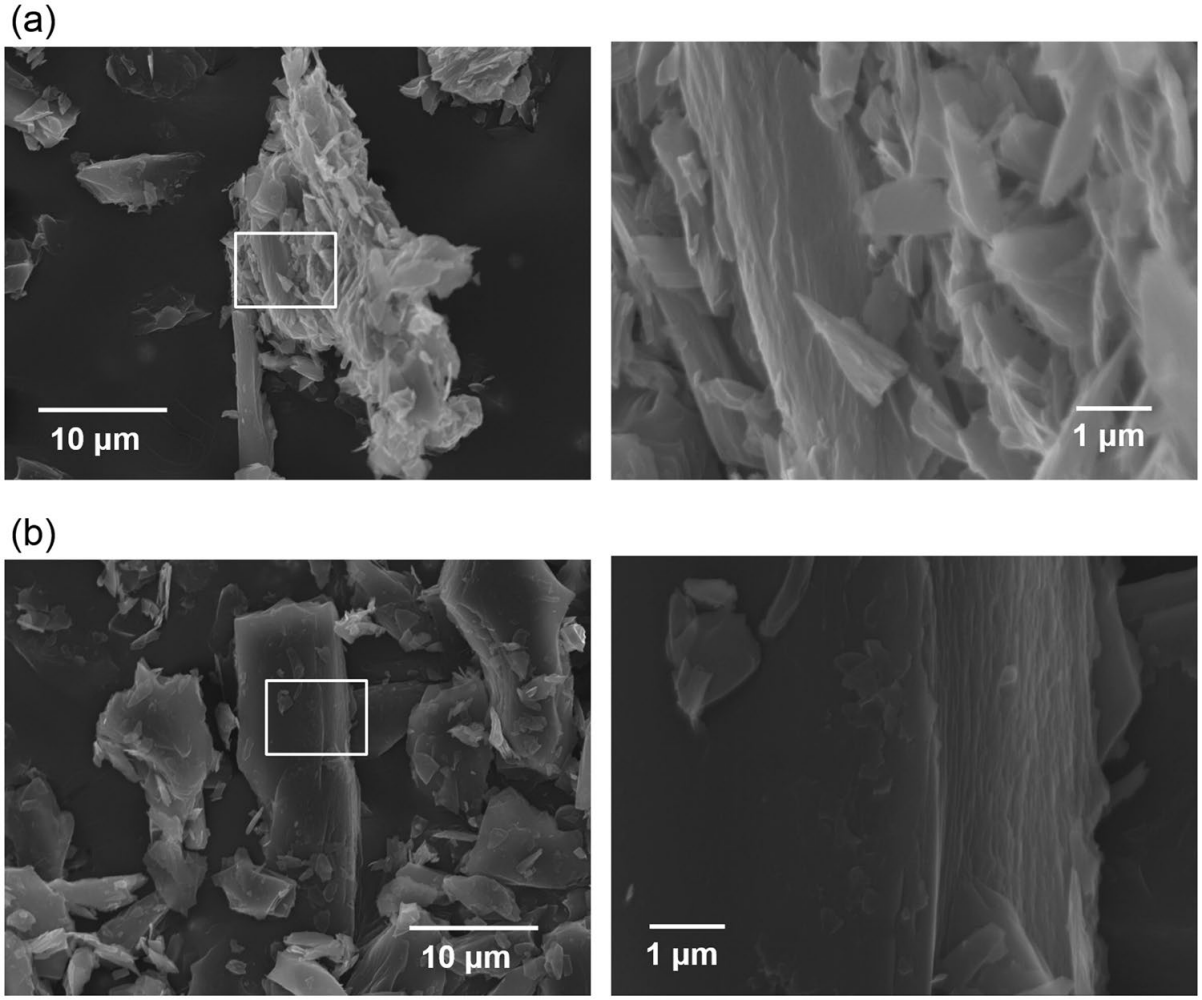
SEM images of activated (**a**) P-GCP and (**b**) P-HOPC with a KOH:precursor ratio of 3.

Scanning electron microscopy (SEM) images of activated P-GCP and P-HOPC samples with a KOH:precursor ratio of 3 are shown in Fig. 7. In the case of P-GCP, most of the images showed flaky structures. However, in the case of P-HOCP, most of the images showed block morphologies. Even when observing the cross-section of the carbon stacking plane, the latter exhibited a cleaner cross-section than the former and retained the ordered structure reflecting the precursor. If P-HOCP could be attacked violently from the isotropic direction, such a structure would not survive. Although it is difficult to directly prove the proposed mechanism, it is sufficient to assume that the two sides have different activation processes.

**Figure 8:**
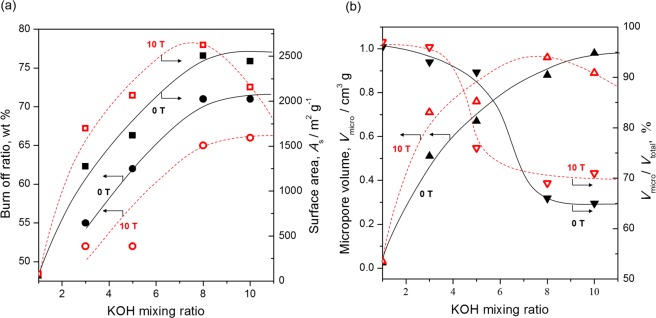
Dependence of (**a**) the burn-off ratio (circles) and surface area (squares) and (**b**) the micropore volume area (upward-pointing triangles) and ratio of micropore volume per total pore volume (downward-pointing triangles) of activated carbon samples prepared from P-GCP (closed marks) and P-HOCP (open marks) on the KOH:precursor ratio.

The relationship between the burn-off ratio and KOH:precursor ratio is plotted in Fig. 8(a). Here, the burn-off ratio, which reflects the progression of activation, was obtained by calculating the weight of carbon lost by comparison with the weight before activation. The burn-off ratio of both P-GCP and P-HOPC increased with the KOH:precursor ratio and then became saturated at KOH:precursor ratios above 8. However, the burn-off ratio of an activated carbon sample prepared from P-HOCP was a few percent lower than that obtained from P-GCP for all KOH:precursor ratios despite using the same activation time of 60 minutes. This result provides evidence that P-HOCP is a less active structure than P-GCP. The surface area is also plotted in Fig. 8(a). The surface area of samples prepared from P-GCP showed the same trend as the burn-off ratio and theoretically increased incrementally with increasing KOH:precursor ratio^30^. Conversely, a decreased surface area was observed at a high KOH:precursor ratio using P-HOCP, which is contrary to the trend in the burn-off ratio. This result shows that the formation of relatively large micropores caused by chemical activation was suppressed and that the micropores also disappeared, which corresponds to the pore distribution results for activated P-HOCP, as shown in Fig. 5(b). This result can be thought to arise from the existence of crystallites at a high density, which inhibited the increase in the pore size beyond a certain size. The increase in the (002) peak intensity of P-HOCP showed an increase with the number of crystallites per unit volume, which has been reported in our previous study^17^. The increase in the crystallite density of P-HOCP was also directly confirmed by transmission electron microscopy (TEM) (Fig. S3). The true density of P-HOCP observed in 1-butylalcohol was also certainly increased by several present over that of P-GCP (Fig. S4). Here, the true density value is not simply related to the number of crystallites. Both P-GCP and P-HOCP contain amorphous carbon and crystallites, and the proportions of these species were only different in each sample; therefore, their density will not differ greatly. As the crystallite density increased with the application of magnetic fields, a large number of micropores having the same distribution were obtain by the progression of activation under the optimum activation condition of around a KOH:precursor ratio of 3.

The micropore volume was almost completely dependent on the tendency of the surface area, as shown in Fig. 8(b), owing to the Type 1 adsorption isotherm. However, as shown on the right axis of Fig. 8(b), the optimum KOH ratio differed between the cases in which fine micropores and relatively large micropores were formed. The rate of increase in the number of fine micropores was the largest at a KOH:precursor ratio of 3. However, the relationship between fine micropores and relatively large micropores was reversed at a KOH:precursor ratio of 5. If relatively large micropores are required, a combination of P-HOCP and a KOH:precursor ratio of 5 was recommended. It seems that the selectivity for pore size can be improved by combining P-HOCP with an appropriate amount of KOH.

## Conclusion

We demonstrated the effect of an applied magnetic field on the preparation of carbon materials. The adsorption ability of an activated carbon sample prepared under a magnetic field was larger than that of sample prepared without a magnetic field by approximately 35%. The most appropriate temperature for carbonization under an applied magnetic field was approximately 800 K; this temperature is where the mesophase of carbon materials appeared. The magnetic field effect on carbonized materials under an unreactive atmosphere was explained by the magnetic orientation in the mesophase, which resembled a liquid crystal. This magnetic field effect disappeared with stabilization treatment. The magnetic field effect on the precursor resulted in the construction of a large number of crystallites, which led to the formation of a large number of micropores. Although the activation of precursors prepared under a magnetic field is difficult, pore formation was effectively performed by the same activation method as that of HOPG. A KOH:precursor ratio of 3 was most suitable for the formation of micropores, and the pore size distribution in samples prepared from P-HOCP was sharper than that in samples prepared from P-GCP. The micropore capacity of activated carbon was improved by preparation under a magnetic field, and we can expect a molecular sieve effect arising from the sharper pore size distribution. Hence, the magnetic field effect can be obtained at a relatively low temperature in the carbonization region, so there is no need to carry out activation at high temperature under a magnetic field, which is very important from an application point of view. These results may also lead to the property control of other carbon materials, such as graphite, because the activated carbon precursor used in this study is optimal for graphite fabrication. Furthermore, many materials other than carbon are categorized as having a negative magnetic susceptibility, which are termed non-magnetic materials; thus, magnetic orientation achieved using this effective procedure can be used to control the properties of such materials.

## Materials and Methods

### Preparation

Coal tar pitch (2.0 g) with a softening point at 553 K, provided by Ad’all Co., Ltd. (Uji, Japan), was heat-treated roughly by two kinds of processes, process A and process B (see Fig. S1(a)). Process A was based on the general method for preparing a graphite precursor. In process A, only the carbonization treatment was carried out with the temperature varied from 553 K to 973 K. Process B was based on the general method for preparing an activated carbon precursor. In process B, stabilization was carried out before carbonization with the time varied from 0 to 120 minutes. The temperature was raised at a rate of 4 K/min. The stabilization process and carbonization process were conducted in atmospheres of air and nitrogen (N_2_), respectively, with a flow rate of 500 sccm. A magnetic field was applied the whole time during heating for process A. Although no magnetic field effects on the stabilization process were observed in our previous study^17^, we applied a magnetic field from time *t*_b_ in process B in the present study to examine the effect of the magnetic field on the carbonization process.

Chemical activation was carried out as shown in Fig. S1(b) using carbonized coal pitch washed by pyridine. The maximum temperature of chemical activation was 1073 K and was maintained for 60 minutes. The ground precursor was immersed in potassium hydroxide (KOH) aqueous solution (the volume ratio of KOH to precursor was 1, 3, 5, 8, or 10) for 3 h before being placed in a furnace. N_2_ gas was flowed at a rate of 500 sccm. The activated sample was washed by enough water.

### Furnace system

An electric furnace system was constructed in the bore (diameter ϕ = 100 mm) of a superconducting magnet (HF-10-100 VHT-4, Sumitomo Heavy Industries, Ltd.), as shown in Fig. S2(a). A ceramic bobbin with a length of 600 mm, wound with Kanthal (Fe-Cr-Al) wire, was housed in a stainless-steel vessel covered with a non-magnetic water cooling jacket and tightly fixed to a 10-T superconducting magnet. A quartz or ceramic tube with an inner diameter of 23 mm and length of 1000 mm was inserted into the furnace bore with an inner diameter of 30 mm. Applying a maximum direct current (DC) voltage of 100 V to the furnace provided an output power of 1520 W and a temperature of 1523 K. The temperature distribution in the quartz tube inserted into the furnace at 798 K in the centre is shown in Fig. S2(b). A uniform temperature of 798 K was maintained with an error of ±5 K. The magnetic field distribution at 10 T in the centre is also presented in Fig. S2(b), which demonstrates the relationship between the furnace and magnet. The temperature at the sample position, which coincided with the centre of the magnetic field, was controlled by a proportional-integral-differential (PID) thermocontroller (E5CN-R2HBT, Omron Co.) with a K-type thermocouple.

### Strictures and properties

The microscopic structures of the prepared carbon materials were characterized by powder X-ray diffraction (XRD) measurements on a Multiflex diffractometer (Rigaku; CuKα radiation). The X-ray source was operated at 40 kV and 20 mA. Samples were scanned at a rate of 4°/min at 0.02° intervals over the range of 2° ≤ 2*θ* ≤ 80°.

The macroscopic structure of the carbonized coal tar pitch was confirmed by an epi-type polarizing microscope (BX51P, Olympus Co.), Photographs of the wide view and magnification view were observed with objective lenses of 5× and 20×, respectively.

The morphology of the prepared activated carbon was observed by a JEOL JSM-7600F scanning electron microscope (SEM).

The adsorption behaviour of the prepared activated carbon samples was determined from N_2_ adsorption isotherms measured by using an in-house gravimetric apparatus at 77 K. Prepared samples were ground and preheated at 393 K and 1 mPa for 3 h before observation of the adsorption isotherms. Total pore volumes were estimated from the adsorption isotherms. The obtained isotherms were also analysed to provide information about the relative surface area, pore diameter, and micropore volume through BET plots, *t*-plots, and Dubinin-Radushkevich (DR) plots, respectively. Pore distributions were analysed by the Horvath–Kawazoe method.

## Supplementary information

Supplementary Information
