# Supplementary material for: Improving the Micropore Capacity of Activated Carbon by Preparation under a High Magnetic Field of 10 T
Source: Sci Rep. 2019 May 16;9:7489. doi: 10.1038/s41598-019-43818-y (PMC6522527; doi:10.1038/s41598-019-43818-y)
Supplement: Supplementary file 1 — Supplementary Information [file 41598_2019_43818_MOESM1_ESM.docx]

**Improving the Micropore Capacity of Activated Carbon by Preparation under a High Magnetic Field of 10 T**

Atom Hamasaki*, Ayumi Sakaguchi, Yuya Sekinuma, Kazuki Fujio, Masashi Iide, Sumio Ozeki

**Supplementary Information**


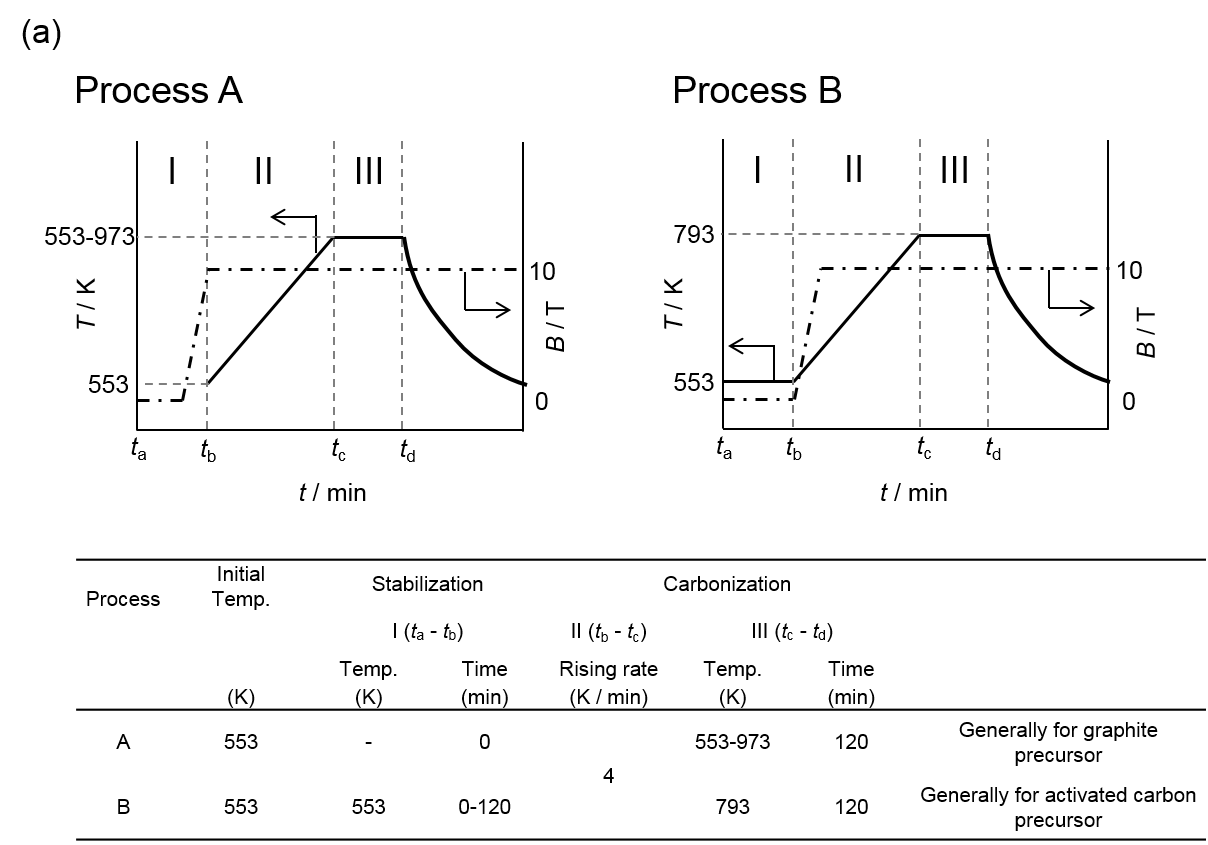


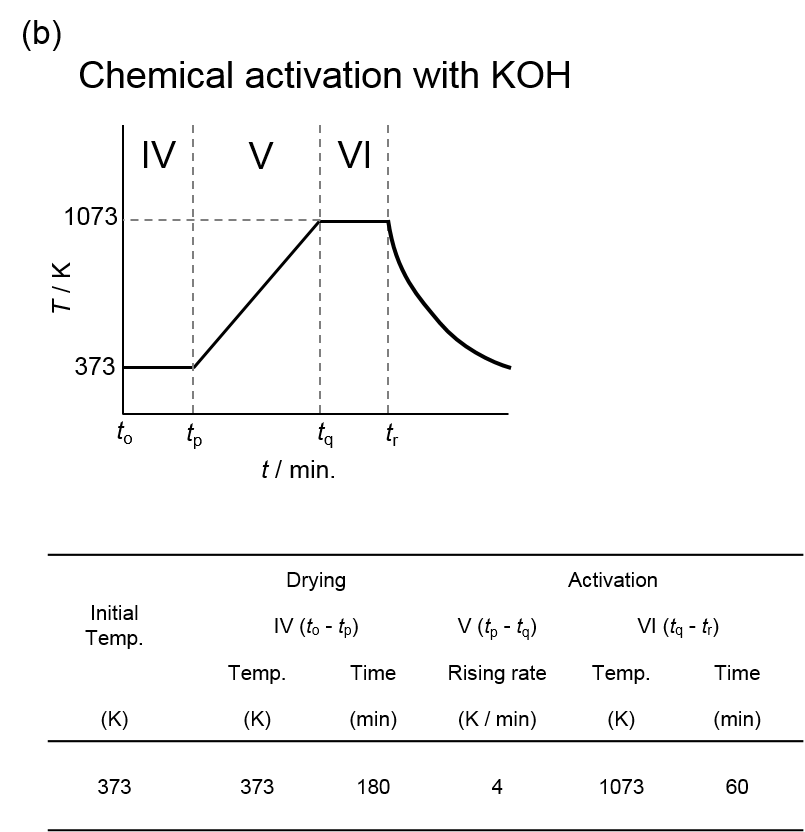


Figure S1. Thermal treatment processes of (a) stabilization and carbonization processes and (b) activation processes.


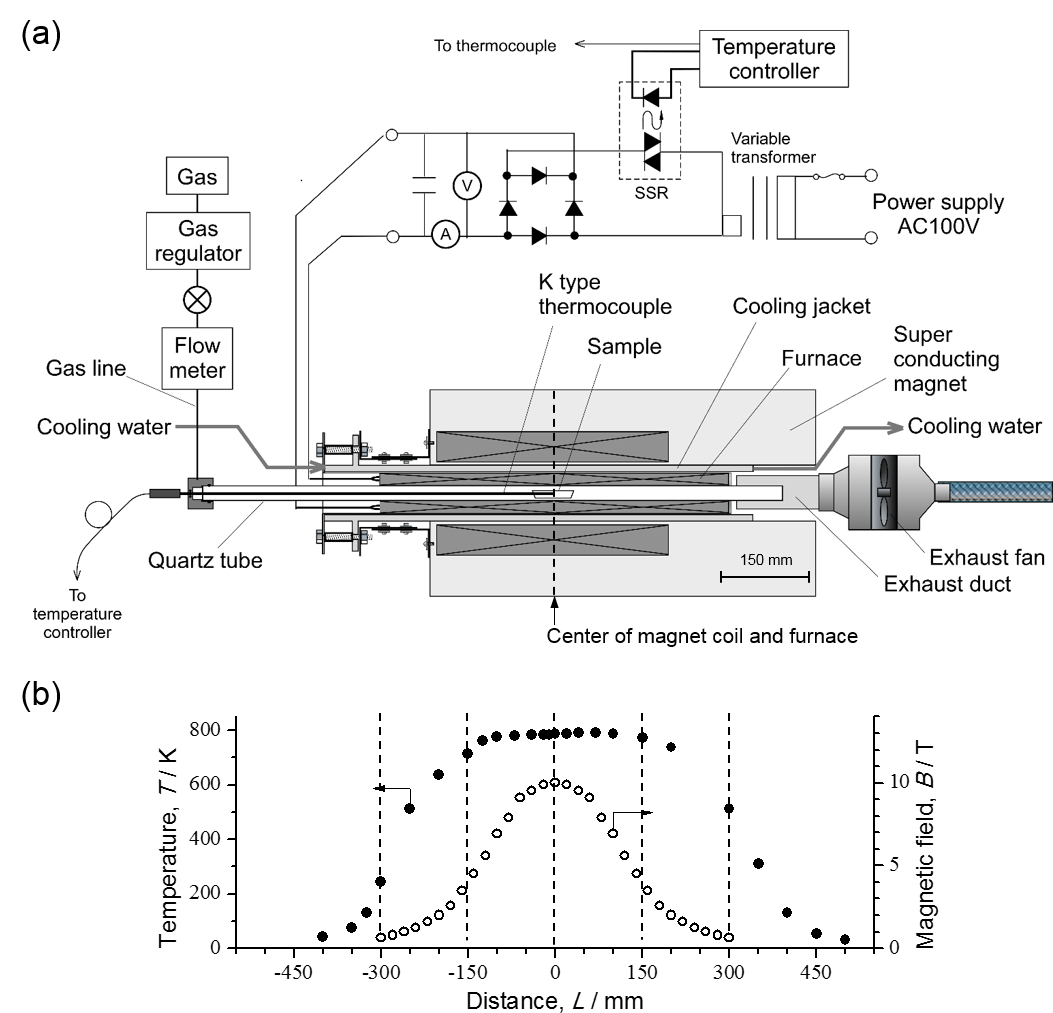


Figure S2. (a) Schematic of the constructed electric furnace system and (b) obtained distributions of temperature (closed circles, left axis) and magnetic field (open circles, right axis).


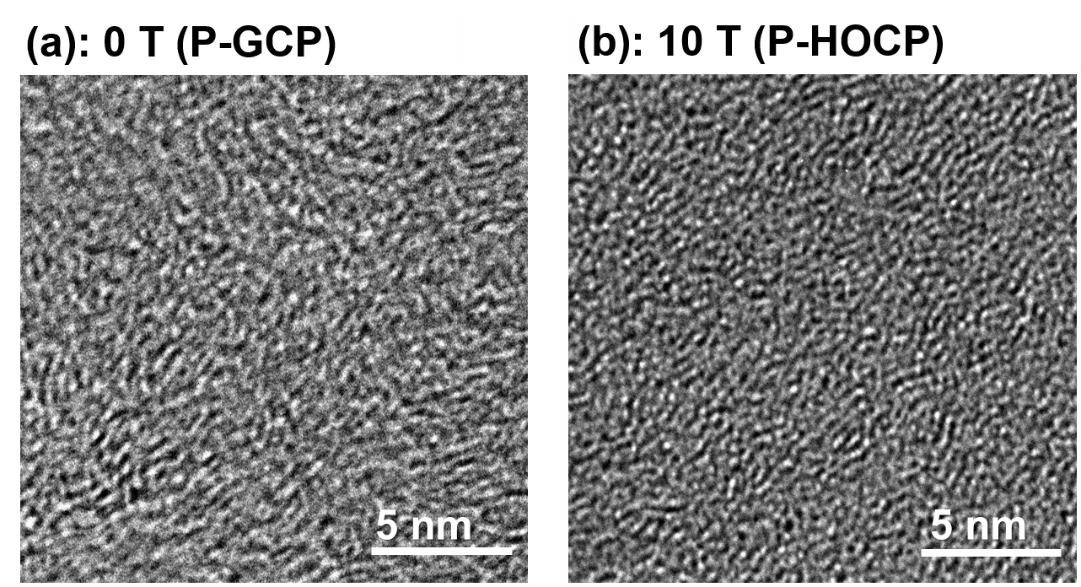


Figure S3. TEM images of prepared (a) P-GCP and (b) P-HOPC samples.


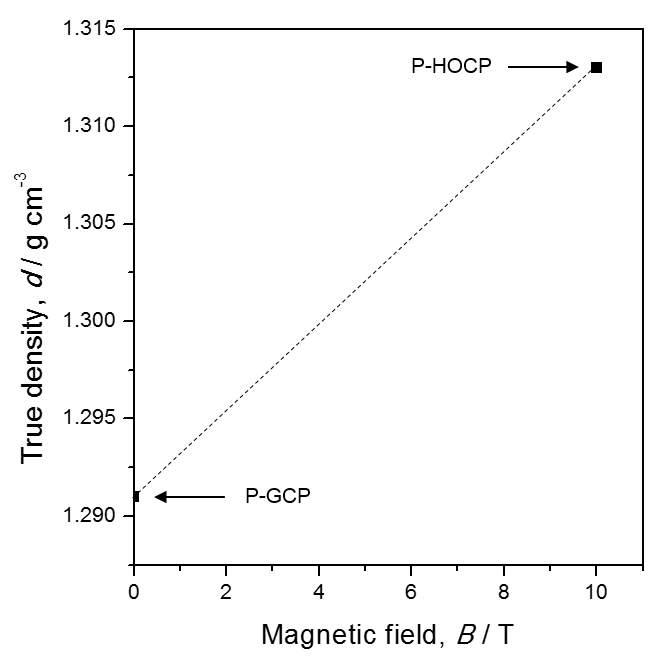


Figure S4. True density of prepared P-GCP and P-HOCP samples.
